# Oral medicinal cannabinoids to relieve symptom burden in the palliative care of patients with advanced cancer: a double-blind, placebo-controlled, randomised clinical trial of efficacy and safety of 1:1 delta-9-tetrahydrocannabinol (THC) and cannabidiol (CBD)

**DOI:** 10.1186/s13063-020-04541-6

**Published:** 2020-07-06

**Authors:** Janet Hardy, Alison Haywood, Gauri Gogna, Jennifer Martin, Patsy Yates, Ristan Greer, Phillip Good

**Affiliations:** 1 Mater Health Services, Mater Research Institute-University of Queensland, Brisbane, Australia; 2 School of Pharmacy, Menzies Health Institute Queensland, Griffith University, Gold Coast, Australia; 3 Mater Research Institute, University of Queensland, Brisbane, Australia; 4 Greenslopes Private Hospital, Brisbane, Gold Coast Health Service, Gold Coast, Australia; 5 The Australian Centre for Cannabinoid Clinical and Research Excellence (ACRE), Newcastle, Australia; 6 Centre for Drug Repurposing and Medicine Research, University of Newcastle, Callaghan, Australia; 7 School of Nursing, Queensland University of Technology, Brisbane, Australia; 8 Centre for Palliative Care Research and Education, Queensland Health, Brisbane, Australia; 9 Mater Health Services, Mater Research Institute-University of Queensland, St Vincent’s Private Hospital, Brisbane, Australia

**Keywords:** Cannabis, Cannabidiol, Cancer, Symptom control, RCT, Palliative care, THC

## Abstract

**Background:**

Despite improvements in medical care, patients with advanced cancer still experience substantial symptom distress. There is increasing interest in the use of medicinal cannabinoids but little high-quality evidence to guide clinicians. This study aims to define the role of a 1:1 delta-9-tetrahydrocannabinol/cannabidiol (THC/CBD) cannabinoid preparation in the management of symptom burden in patients with advanced cancer undergoing standard palliative care.

**Methods and design:**

One hundred fifty participants will be recruited from five sites within the Queensland Palliative Care Research Group (QPCRG) and randomly assigned to an active treatment or placebo group. This study is a pragmatic multicentre, randomised, placebo-controlled, two-arm trial of escalating doses of an oral 1:1 THC/CBD cannabinoid preparation. It will compare efficacy and safety outcomes of a titrated dose (10 mg/10 mg/mL oral solution formulation, dose range 2.5 mg/2.5 mg–30 mg/30 mg/day) against placebo. There is a 2-week patient-determined titration phase, using escalating doses of 1:1 THC/CBD or placebo, to reach a dose that achieves symptom relief with tolerable side effects. This is then followed by a further 2-week assessment period on the stable dose determined in collaboration with clinicians. The primary objective is to assess the effect of escalating doses of a 1:1 THC/CBD cannabinoid preparation against placebo on change in total symptom score, with secondary objectives including establishing a patient-determined effective dose, the change in total physical and emotional sores, global impression of change, anxiety and depression, opioid use, quality of life and adverse effects.

**Discussion:**

This will be the first placebo-controlled clinical trial to rigorously evaluate the efficacy, safety and acceptability of 1:1 THC/CBD for symptom relief in advanced cancer patients. This study will allow the medical community to have some evidence to present to patients wishing to access cannabis for their symptoms caused by advanced malignancy.

**Trial registration:**

ACTRN, ACTRN12619000037101. Registered on 14 January 2019.

Trial Sponsor: Mater Misericordiae Limited (MML) and Mater Medical Research Institute Limited (MMRI)—Raymond Terrace, South Brisbane, Brisbane, QLD, Australia

## Background

Despite improvements in medical care, patients with advanced cancer still experience substantial symptom distress [1]. Palliative care aims to take a skilled, holistic approach to improve patients’ symptoms and quality of life. Whilst there is a range of analgesic medication available for pain management, the control of many other symptoms (e.g. fatigue, anorexia, weight loss) remains a challenge [2].

There has been increasing interest in the use of medicinal cannabinoids over recent years, particularly for the relief of symptoms in palliative care patients [3]. Recent legislative change in several Australian states provided pathways for the use of medicinal cannabinoids for a range of indications including chemotherapy-induced nausea and vomiting (CINV), resistant epilepsy, pain and spasticity in multiple sclerosis, and symptoms associated with terminal illness [4].

Cannabis contains almost 500 bioactive compounds, including over 70 different cannabinoids [5]. The predominant cannabinoids include delta-9-tetrahydrocannabinol (THC) and cannabidiol (CBD). THC is the main psychoactive component of cannabinoids and is thought to act as a partial agonist on the endocannabinoid system. Potential benefits of THC include analgesia, anti-nausea and muscle relaxation, with potential side effects including intoxication, psychosis, anxiety and sedation. The recommended dose range for oral THC varies from 2.5 to 40 mg/day [6]. In contrast, CBD is not intoxicating and has a range of anxiolytic, antipsychotic, anti-inflammatory, anti-oxidative, anti-convulsant and neuroprotective effects [7]. CBD is also considered to mediate many of the adverse psychotropic effects of THC, although this research is still emerging [8]. CBD has been used with a dosing in the range of 40 to 1280 mg/day orally [9].

There is little high-quality evidence of benefit to date for the use of medicinal cannabinoids. The most recent review from the USA National Academies of Sciences, Engineering, and Medicine found substantial evidence for the use of medicinal cannabinoids for treatment of some types of chronic pain, CINV and spasticity in multiple sclerosis in some patients, with moderate evidence for sleep disorders [10]. Cannabinoid products are licenced for a range of conditions in different countries, with little consistency between countries for indication and dosing [11]. Whilst cannabinoids may have clinical indication, their use is not without potential for substantial harms, and further research is needed to define their role in medical practice [3, 11].

There are many unknowns when it comes to prescribing medicinal cannabis [11]. This includes the formulation of the drug (ratios of THC or CBD), the dosing of the drug and the best route of delivery. Ongoing concerns remain around the uncertainty over the optimal formulation, toxicity and abuse potential. The 1:1 THC/CBD ratio used in formulations may deliver sufficient CBD to ameliorate the psychotoxic effects of THC but is unlikely to produce significant CBD therapeutic effects.

To date, there has been no formal examination of the effect of different THC/CBD ratios. We will therefore compare efficacy and safety outcomes of a 1:1 THC/CBD formulation against placebo, with doses of both THC and CBD escalated to doses previously shown to be safe.

## Methods

### Aims and objectives

This study aims to define the role of cannabinoids in the management of symptoms in patients with advanced cancer, who are receiving standard palliative care. The hypothesis is that medicinal cannabinoids will reduce the total symptom burden in these patients compared to placebo.

The primary objective is to assess the effect of escalating doses of a 1:1 THC/CBD cannabinoid preparation against placebo on total symptom score at day 14 as measured by the Edmonton Symptom Assessment Scale (ESAS) (change in score from baseline). Secondary objectives are to establish a patient-determined effective dose of a 1:1 THC/CBD formulation and assess the effect on symptom scores at days 7, 21 and 28. Other secondary objectives include assessing the change in total physical and emotional sores, global impression of change, anxiety and depression, opioid use and quality of life at the same time points and to document adverse effects associated with cannabinoid use.

### Study design

This study is a pragmatic multicentre, randomised, placebo-controlled, two-arm parallel trial of escalating doses of oral THC/CBD 10 mg/10 mg/mL oral solution. There is a 2-week patient-determined titration phase, using escalating doses of a 1:1 THC/CBD combination or placebo, to reach a dose that achieves symptom relief with tolerable side effects. This is followed by a further 2-week assessment period on the stable dose determined in collaboration with clinicians.

Eligible patients will be randomly assigned in equal numbers to one of the two study arms. Randomisation schedules will be developed for each site using random number tables, computer generated at an independent centre. Treatment for each patient will be allocated according to a permuted block randomisation schedule held by the central registry. Block randomisation within each centre will ensure even allocation to each arm in each site. The pharmacy will be notified of a participant, and a completed script with the participant’s study ID number will be given to the site pharmacy. The pharmacist will randomise the participant according to the schedule and dispense the medication in a labelled bottle. The participant ID, allocation number, date of request, preparation and dispensing will be recorded in a log maintained by the site pharmacist for each randomisation.

All participants, caregivers, investigators and clinical staff will remain blind to study assignment until trial completion. The code will only be broken in cases of a clinical emergency. An investigator not directly involved in the randomisation of patients will keep an un-blinded copy of the randomisation schedule and will be contacted in the event of the need to un-blind. All study drugs and placebo will be in oil solution form and identical in appearance. All oil solutions will be matched for taste, colour and bottle size to preserve the blinding irrespective of the contents; each participant will receive the oil solution in a prepacked bottle labelled with their individual trial participation ID number and consecutively numbered according to the randomisation scale.

### Intervention

Cannabinoid oils, supplied by LG Pharma, will be used in this study. The decision to use oils was based on a number of factors including product availability, ability to access ongoing supplies, ability to produce matched placebo, regulations surrounding the production and transport of cannabinoid products and cost.

Arm 1 of the trial consists of THC/CBD 10 mg/10 mg/mL oral solution (LG Pharma Pty Ltd) with a dose range of 2.5 mg/2.5 mg–30 mg/30 mg/day and arm 2 a matching placebo oral oil solution. Participants are asked to take daily doses as per dosing schedule instructions. The study drug is to commence on the day of baseline assessment, continuing for a maximum of 28 days.

The cannabinoid oils and placebo will be dispensed in identical 50-mL bottles containing a sufficient quantity of oil. Bottles are to be stored at room temperature (25 °C) until used and stored away from light. The product will be dispensed from bottles with a wadded child-resistant cap. Participants will be given replacement bottles to complete the study as required. Participants will be educated on how to administer daily doses using supplied 1-mL syringes appropriate for the dose. The participant will complete a daily dosing schedule diary at home to record medication taken. Cannabinoid oil that has been allocated to a participant and not used will be returned to the research nurse/research officer and destroyed by the local pharmacy as per pharmacy guidelines.

Dose titration will be confirmed following regular consultation. Each participant will be advised to increase their dose according to a schedule every 2 days until they are satisfied with their symptom improvement or they experience unacceptable side effects. Dose titration downwards will also be allowed, in consultation with research staff. The participant in collaboration with research staff will define the dose level at which they will continue until the primary outcome point (14 days). Participants will then be given the option of remaining on the blinded oil solution for a further 2 weeks (28 days total) for continuing assessment of efficacy and adverse events (continue final dose if perceived to be of benefit and tolerated). Assessment at 28 days following 14 days of stable dose is a secondary end point.

### Study setting

Participants will be recruited from five sites within the Queensland Palliative Care Research Group (QPCRG). These five sites have been chosen from the QPCRG as they have an established research team including research support systems within Palliative Care and have previous experience in trial participation in this setting. It is anticipated that this will be predominately an outpatient study, with these sites having the ability to access this participant population from their established clinic service.

All participants will be given standard palliative care according to the local practice of the recruiting centre [12]. They will continue all current medications including opioids, antiemetics, sedatives and specific anti-cancer therapy (including chemotherapy, immunotherapy and radiotherapy).

### Study participants

Patients who meet the inclusion criteria will have an advanced histologically proven cancer diagnosis (metastatic or locally advanced), be known to and be receiving palliative care at the recruiting centre and have an ESAS Total Symptom Distress Score (TSDS) of ≥ 10/90 for cancer-related symptoms and at least one individual ESAS score ≥ 3/10 [13]. They must have a Performance Status AKPS (Australia-modified Karnofsky Scale score) of ≥ 30/100 [14], be aged ≥ 25 years, have a negative THC urine test at commencement of trial and be able to tolerate oral medications and must be either English-speaking or have an interpreter available.

Female participants must have a negative pregnancy urine test at eligibility (only if of reproductive potential) and agree to avoid pregnancy during the study and 12 weeks following the last dose of the study drug. Males must agree to avoid fathering a child and to not donate sperm during the study and for at least 12 weeks following the last dose of the study drug. Further to this, the Alcohol, Smoking and Substance Involvement Screening Test (ASSIST) will be completed [15]. It is designed to determine whether harmful substances are being used and go undetected or become worse. The assessment is comprised of 8 questions assessing, tobacco, alcohol, cocaine, amphetamine-type stimulants, sedatives, hallucinogens, opiates and ‘other drugs’.

Exclusion criteria include a history of hypersensitivity to any cannabinoid, unstable untreated cardiovascular disease, severe hepatic impairment (total bilirubin ≥ 1.5 times the upper limit of the normal range), aspartate aminotransferase (AST) and alanine aminotransferase (ALT) ≥ 3.0 times the upper limit of the normal range (subjects with liver metastasis may have an AST and ALT of ≥ 5.0 times the upper limit of normal), severe renal impairment (eGFR ≤ 20 mL/min/1.73 m^2^), a history of psychiatric disorders (severe depression or anxiety, personality disorder, psychosis, schizophrenia, first-degree relative with schizophrenia and/or suicidal ideation), cognitive impairment (St Louis University Mental Status examination (SLUMS) ≤ 20/30) [16] and a known substance use disorder (Alcohol, Smoking and Substance Involvement Screening Test (ASSIST) examination scoring > 27) for any substance.

Potential participants will be excluded if they have a history suggesting that drug diversion may be a risk for them or their family/carers, have participated in a trial of a new clinical entity within the last 28 days or had treatment with a new specific anti-cancer agent (chemotherapy, targeted or hormonal therapy) or radiation within the last 7 days.

### Consent process

The process for obtaining consent for this study will be exchanging information between the study staff and potential participants and any other person the participant wishes to include in the discussion. A participant information sheet (PICF) will be provided in written form and will be used as the basis for the discussion. This will cover the purpose, expected procedures, participant requirements, risks, benefits, burdens and side effects that are expected or possible during the study. Participants are specifically informed that due to the nature of this medication and current laws in Queensland, you will NOT be able to drive or operate heavy machinery whilst taking the medication. Potential participants will be given the opportunity (in time and physical capacity) to consider the study and formulate any questions. All questions will be addressed and answered fully. An actual time point for consent will not be specified as this will be determined by the person’s physical condition. The consent form is to be completed by trained study team members in accordance with the requirements of the approving ethics committee. The form is to be signed and dated by the participant.

### Assessments

Participants will receive 2 times/weekly research nurse phone calls in the first 2 weeks and outpatient clinic medical review visits at days 7, 14, 21 and 28, with outcome measures recorded at these points.

Symptom burden will be measured using the Edmonton Symptom Assessment Scale (ESAS). Confirmed documented disease status will be assessed at days 14 and 28, where applicable.

A routine haematology and biochemistry screen including liver function tests will be taken at eligibility/baseline assessment. Blood for C-reactive protein (CRP) as a basic test of inflammation will be taken at baseline, day 14 and day 28. All consenting participants will have a urine test to confirm nil recent use of THC-related products as a pre-screen. Female participants of childbearing potential will have a urine test to determine pregnancy. At day 14, a urine sample will be collected and stored (frozen) until the completion and un-blinding of the study. Samples will be stored on site and transported to central storage in batches over the course of the study. These samples will be for post-trial analysis for evidence of no THC product use during the trial.

Participants will be contacted at day 56 (+ 4 weeks post last dose) for the purposes of safety follow-up and recording of post-study cannabinoid use. Date of death will be recorded for all participants up to the census point. This protocol has been written in accordance with the Standard Protocol Items: Recommendations for Interventional Trials (SPIRIT) (Table 1).

**Table 1:**
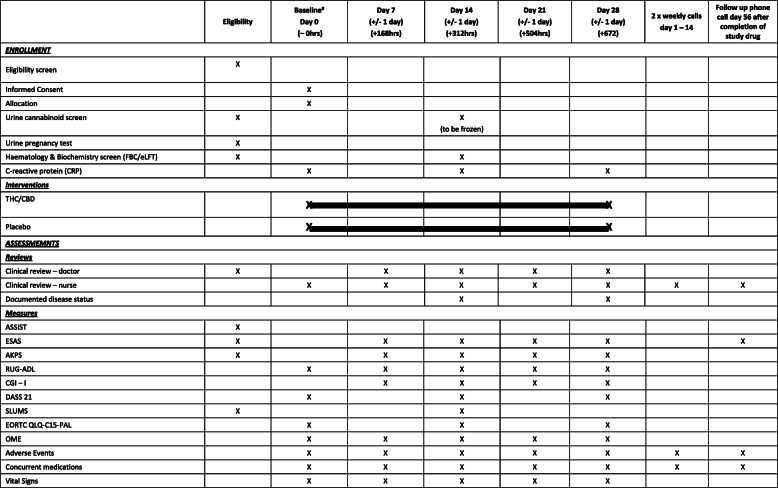
SPIRIT figure—study schedule

### Outcomes and assessment tools

The primary outcome is the change from baseline of total ESAS TSDS at day 14.

The ESAS is a 9-item inventory rated on an 11-point scale anchored at 0 (no problem) to 10 (worst problem). It assesses both physical and psychological symptoms, plus general wellbeing. It has been validated in the assessment of symptoms in cancer patients [13].

Secondary outcomes include:

1. Patient-determined effective dose of THC/CBD formulation, defined as the dose that achieves symptom relief with acceptable side effects.
2. ESAS TSDS at days 7, 21 and 28.
3. Physical and emotional ESAS scores at each time point.
  Physical scores will be measured by the Australia-modified Karnofsky Performance Status (AKPS) and Resource Utilisation Groups-Activities of Daily Living Scale (RUG-ADL). The AKPS is a validated variant of the Karnofsky Performance Status. The Australian version can be applied to both in- and outpatients and is sensitive to changes in function over time [14]. The RUG-ADL is an instrument developed for the measurement of nursing dependency. The ADL scale measures patients’ needs for assistance in activities of daily living (eating, bed mobility, transferring and toileting) [17].
4. Individual symptom scores (descriptive analysis only).
5. Oral morphine equivalent (OME), average use at baseline and weekly.
6. Patient Global Impression of Change (PGIC), days 7, 14, 21 and 28.
  This is a subjective measure of symptom change completed by the patients themselves [18].
7. Clinical Global Impressions (CGI) Scale, days 7, 14, 21 and 28.
  This is a subjective measure of symptom change completed by the investigating clinician [18].
8. The Depression, Anxiety, Stress Scale (DASS-21) score baseline and days 14 and 28.
  DASS-21 is a self-reported 21-item scale, 7 questions per sub-item questionnaire measuring depression, anxiety and stress. The DASS-21 will be used for assessment at baseline (day 0) and days 14 and 28 [19].
9. EORTC score QoL baseline and days 14 and 28 [20].
  This is a QoL measure found valid for use in a wide variety of cancer populations. To reduce patient burden, we will use the 15-question subset commonly used for the palliative care population.
10. NCI common terminology for adverse events V4.03, days 2, 4, 7, 9, 11, 14, 16, 18, 21, 23, 25 and 28 [21]
  The NCI CTCAE is a severity grading system for adverse events. Adverse events relate to an unfavourable or unintended sign, symptom or disease temporarily associated with a medical treatment or a procedure which may not be related to the medical treatment or procedure. The CTCAE has a grading of 1 (mild) to 5 (death). The known common adverse events associated with cannabinoids are confusion, somnolence, paranoia, anxiety, mood changes, psychosis, hypertension, tachycardia, hyperhidrosis, nausea, vomiting and abdominal pain [21].

The study will assess adverse events (AE) and serious adverse events (SAE) using the criteria of the NCI CTCAE V4.0 [21]. The known common AEs associated with THC/CBD will be specifically addressed at each time point. Finally, a detailed concurrent medication list is to be updated and recorded at baseline (day 0) and days 7, 14, 21 and 28. This is to include over-the-counter medications, prescribed medications and complementary medication.

### Statistical analysis and sample size

Allowing 20% for attrition, and with improvement of ≥ 6 for arm 1 compared to placebo, it is anticipated that 150 participants (75 per arm) should be randomised to achieve a sample size of 60 participants per arm, assuming 80% power, a 1:1 randomisation scheme and a type 1 error of 5% (two-tailed), and a standard deviation of 11.6. The sample size is based on previous work by Hui et al. [13] who determined the minimal clinically important difference in the TSDS to be 5.7 [13]. As such, we have elected to use an improvement of the TSDS of ≥ 6 as a clinically significant change. Stata software (StataCorp. 2013. Stata Statistical Software: Release 13. College Station, TX: StataCorp) was used to estimate the sample size.

The superiority of arm 1 compared to placebo will be tested by comparing the response to each arm after 14 days, relative to baseline.

Descriptive analyses and frequency distributions will be generated from participants’ demographic and clinical characteristics, with all variables explored using graphical methods and summary statistics. For the primary outcome, generalised estimating equations with the appropriate link function will be developed to assess the effect of treatment adjusted for centre. For secondary outcomes, where measurements from individual subjects are recorded several times over the course of the study, change over time in the various secondary outcome measures will be assessed using mixed models, accounting for within-subject correlation and effects due to centre. For all statistical analyses, any influence of potential covariates, for example age, sex, type of cancer and disease severity, will be explored. This study is powered to detect superiority of arm 1 over placebo.

An interim analysis will be performed after 50% participants have completed 14 days of the trial. The analysis will be performed by an independent biostatistician blinded for the treatment allocation and reported to the investigators and the Data Safety Monitoring Board (DSMB). The purpose of the interim analysis is primarily to monitor and ensure safety of participants rather than evidence of such benefit that early stopping of the trial is justified. AEs and SAEs will be stratified by type and severity. The frequency of AEs and SAEs will be compared between treatment groups using chi-square tests and logistic regression if indicated to adjust for any baseline differences between groups. Differences in baseline characteristics are not anticipated, as the randomisation process should ensure that the patient groups are similar at baseline. However, as the numbers at interim analysis will be relatively smaller, it is possible that differences may arise by chance. All baseline characteristics will be assessed and any imbalance addressed in the analysis.

If the interim analysis shows a significant difference, the investigators and DSMB will be un-blinded to the study groups and make any stopping decision on the basis of the nature of any AEs and/or SAEs and ethical grounds, as well as consideration of any statistical differences between the groups. Grounds for stopping on evidence of clear benefit will be considered. The Peto approach will be taken with symmetric stopping boundaries at *p* = 0.001.

A detailed statistical analysis plan will be prepared and ratified by the DSMB. Stata, SAS (SAS® version 9.4 software (SAS Institute Inc., Cary, NC, USA)) or R (R Core Team (2017). R: A language and environment for statistical computing. R Foundation for Statistical Computing, Vienna, Austria. URL https://www.R-project.org/) will be used for analysis and all code recorded for the purposes of ensuring reproducible research.

### Data collection and management

Data will be sourced from a number of modes. The study is mainly based in the outpatient clinic, so most data will be collected from the participant and recoded in the corresponding CRF or questionnaires. Some data will be collected from medical records, but the majority of the data will be collected from:

**Table:** 

Measure	Source	Collected by
General medical information	Clinical record	Medical officer
General demographic data	Clinical record	Study nurse
Pathology results—blood	Pathology report	Pathology
Pathology results—urine	Clinical record	Study nurse
Vital signs	CRF/clinical record	Study nurse
Concurrent medications/OME	Clinical record	Study nurse
Study trial data—questionnaires—ASSIST, SLUMS, EORTC QLQ-C15-PAL	CRF	Patient, study nurse, medical officer
Study trial data—ESAS, AKPS, RUG-ADL, PGIC, CGI-S/I, DASS-21	CRF	Patient, study nurse, medical officer
Side effects—safety	CRF, clinical record	Study nurse, medical officer

All data collected will be kept in a patient file (identified by ID number only). All data will be stored in a locked filing cabinet. At completion of the study, all CRFs will be collated and archived. Electronic files will be password protected and held within a locked office. All patient files will be reconciled and stored along with all study materials, both hard copy and electronic, consistent with the regulations of the hospital regarding the retention and disposal of patient records.

The trial will be conducted with permission and in accordance with Queensland Health regulations on the use of medical cannabis and subject to approval and monitoring by each clinical site’s HREC [4, 22]. An independent DSMB to include a statistician, clinical pharmacologist, palliative care specialist and consumers will be formed and will meet regularly, with primary responsibility for monitoring adverse and serious adverse events. All AE’s and SAE’s will be reviewed at a minimum of 6 monthly intervals, or more frequently if needed.

### Post-trial care

There is no anticipated harm or compensation for trial participation. Participants may be able to access ongoing cannabinoid products through entry into other open-label studies but this is not guaranteed. All clinical investigators will be approved authorised cannabis prescribers and will be able to continue to prescribe, as long as the participant can fund their own supply. Participants will be informed of approved products and suppliers. The dose and formulation used post study will be at the discretion of the patient and prescriber.

## Discussion

The use of cannabis for symptom control continues to be a topical issue within medicine, and this study is the first placebo-controlled, randomised trial to assess the efficacy of cannabinoids in advanced cancer patients. A major strength of this study is that it will target symptom burden as a whole, rather than just individual symptoms, in an attempt to capture the improvement in general wellbeing reported anecdotally by many who have used cannabis [23, 24]. Randomisation with placebo is essential because of the well-documented overreporting of benefit in uncontrolled trials and high placebo response rates in cancer pain trials [25, 26]. The trial design is pragmatic, intended to allow as wide a range of participants as possible, to enable the results to be as real-world as possible. This study also allows for some data on dosing and formulation of THC/CBD specifically.

### Trial status

Trial recruitment started on 09 September 2019 with protocol version number 1.3 (protocol date 30 August 2019). Recruitment is expected to continue until the end of 2021.

## Data Availability

The datasets used and/or analysed during the current study are available from the corresponding author on reasonable request.

## Abbreviations

AE: Adverse events
AKPS: Australia-modified Karnofsky Scale
ALT: Alanine aminotransferase
ASSIST: Alcohol, Smoking and Substance Involvement Screening Test
AST: Aspartate aminotransferase
BD: Twice daily
CBD: Cannabidiol
CGI: Clinical Global Impressions Scale
CINV: Chemotherapy-induced nausea and vomiting
CPMP/ICH/135/95: Note for Guidance on Good Clinical Practice
CRF: Case report form
CRP: C-reactive protein
DASS-21: The Depression, Anxiety, Stress Scale
DSMB: Data Safety Monitoring Board
EORTC QLQ-C15-PAL: European Organisation for Research and Treatment of Cancer
ESAS: Edmonton Symptom Assessment Scale
GCCS: Gold Coast Cancer Services
LGP: Little Green Pharma
GMP: Good manufacturing practice
HREC: Human Research Ethics Committee
ID: Identification
MANE: Morning
MML: Mater Misericordiae Limited
MRFF: Medical Research Future Fund
NHMRC: National Health and Medical Research Council
NOCTE: At night
OME: Oral morphine equivalent
QoL: Quality of life
QPCRG: Queensland Palliative Care Research Group
RUG-ADL: Resource Utilisation Groups-Activities of Daily Living Scale
SAE: Serious adverse events
SLUMS: St Louis University Mental Status exam
TDS: Three times a day
TGA: Therapeutic Goods Administration
THC: Delta-9-tetrahydrocannabinol
TSDS: Total Symptom Distress Score
